# Bridging Recovery Initiative Despite Gaps in Entry (BRIDGE): study protocol for a randomized controlled trial of a bridge clinic compared with usual care for patients with opioid use disorder

**DOI:** 10.1186/s13063-021-05698-4

**Published:** 2021-10-30

**Authors:** David E. Marcovitz, Katie D. White, William Sullivan, Heather M. Limper, Mary Lynn Dear, Reagan Buie, David A. Edwards, Cody Chastain, Kristopher A. Kast, Christopher J. Lindsell, Vanderbilt Learning Health System Investigators

**Affiliations:** Vanderbilt University Medical Center, Vanderbilt Psychiatric Hospital, 1603 23rd Ave South, Nashville, TN 37212 USA

**Keywords:** Pragmatic clinical trial, Substance use disorder, Opioid use disorder, Bridge clinic

## Abstract

**Background:**

Patients with substance use disorders are overrepresented among general hospital inpatients, and their admissions are associated with longer lengths of stay and increased readmission rates. Amid the national opioid crisis, increased attention has been given to the integration of addiction with routine medical care in order to better engage such patients and minimize fragmentation of care. General hospital addiction consultation services and transitional, hospital-based “bridge” clinics have emerged as potential solutions. We designed the Bridging Recovery Initiative Despite Gaps in Entry (BRIDGE) trial to determine if these clinics are superior to usual care for these patients.

**Methods:**

This single-center, pragmatic, randomized controlled clinical trial is enrolling hospitalized patients with opioid use disorder (OUD) who are initiating medication for OUD (MOUD) in consultation with the addiction consult service. Patients are randomized for referral to a co-located, transitional, multidisciplinary bridge clinic or to usual care, with the assignment probability being determined by clinic capacity. The primary endpoint is hospital length of stay. Secondary endpoints include quality of life, linkage to care, self-reported buprenorphine or naltrexone fills, rate of known recurrent opioid use, readmission rates, and costs. Implementation endpoints include willingness to be referred to the bridge clinic, attendance rates among those referred, and reasons why patients were not eligible for referral. The main analysis will use an intent-to-treat approach with full covariate adjustment.

**Discussion:**

This ongoing pragmatic trial will provide evidence on the effectiveness of proactive linkage to a bridge clinic intervention for hospitalized patients with OUD initiating evidence-based pharmacotherapy in consultation with the addiction consult service.

**Trial registration:**

ClinicalTrials.govNCT04084392. Registered on 10 September 2019. The study has been approved by the Vanderbilt Institutional Review Board. The current approved protocol is dated version May 12, 2021.

**Supplementary Information:**

The online version contains supplementary material available at 10.1186/s13063-021-05698-4.

## Background

Patients with substance use disorders (SUDs) are overrepresented among general hospital inpatients [1], and their admissions are associated with longer lengths of stay and increased readmission rates [2, 3]. There is also broad understanding that the majority of patients with SUD do not seek care, falling into the “treatment gap” on epidemiological surveys [4]. The national opioid crisis has brought renewed attention to the question of how to most effectively reach patients struggling with opioid use disorder (OUD) as early as possible in their course of illness in order to reduce morbidity and mortality [5, 6].

Treatment with medications for OUD (MOUD) is known to be effective. Patients treated with MOUD have higher rates of retention in treatment, lower rates of illicit drug use, fewer infectious complications, and reduced opioid overdose deaths than those who do not receive MOUD [13, 14]. Rates of sustained abstinence are less than 10% for patients not treated with MOUD, such as buprenorphine-naloxone [10–12]. However, when persons with OUD engage with the medical system, the availability of evidence-based treatment, including MOUD, is limited [7]. One study reported that less than 8% of patients with injection drug use-related endocarditis were initiated and referred for outpatient treatment including MOUD [8, 9]. The combination of high utilization of medical services and poor access to evidence-based addiction treatment has heightened awareness of the need to integrate addiction treatment into routine medical care.

Efforts focused on improving access to addiction care have historically been divided into inpatient and outpatient efforts, with a more recent focus on the transitions of care between these two settings. Preceding the opioid crisis, efforts to integrate SUD treatment into primary care were underway [15], and more recently, efforts have focused on collaborative care for OUD in the primary care setting as a means to increase the availability of MOUD [16]. For hospitalized patients, general hospital addiction consultation services have now been described at multiple institutions [6, 17, 18] with some studies showing an impact on 30-day abstinence, addiction severity, and readmission [17, 19, 20]. In order to better support such inpatient efforts and link patients into longitudinal care more rapidly, transitional “bridge” clinics have emerged as potential solutions to the problems of care continuum fragmentation, increased length of stay, and high readmission rates [21]. Though these bridge clinics vary in many respects, common features include their transitional nature, low threshold and low wait-time to access, and geographic co-location within the hospital system to which the patient presented.

Though the potential benefits of bridge clinics are many, little is known about the effectiveness of the bridge clinic model. At the patient level, it remains to be seen whether the intervention can effectively retain patients, reduce relapse to illicit substances, and improve medical outcomes alongside quality of life. At the system level, the potential benefits of the bridge clinic model are promising but to date unproven.

One area of potential for significant impact involves hospital admissions for patients with infectious complications of injection drug use. These admissions have been increasing year-over-year [22], with one study in North Carolina estimating a cost per admission of $50,000 for endocarditis (with 42% uninsured or on Medicaid) [23]. Another study involving a safety net hospital described an annual cost of $11.4 million to care for 349 patients with infectious complications of intravenous drug use, in this case with a 90% rate of uninsured or Medicaid status [24]. These national trends were reflected in our own institutional data with a 55% increase in admissions with OUD-related diagnostic codes in the first 6 months of 2018 compared to the year prior. The average length of stay for patients with OUD and need for intravenous antibiotic treatment was 15 days compared to a hospital-wide average of just 3 days. Whether the use of a bridge clinic model improves patient outcomes while reducing resource use remains unknown.

### Objectives

We designed the Bridging Recovery Initiative Despite Gaps in Entry (BRIDGE) trial to test the hypothesis that patients assigned to a bridge clinic intervention will have reduced hospital LOS, reduced readmission rates, improved linkage to care, improved MOUD fill rates, fewer relapses, improved quality of life, and reduced total cost of care compared with patients assigned to usual care.

## Methods and design

### Methods

The bridge clinic was not designed as a research intervention; rather, this prospective study was designed to evaluate the effectiveness of the clinic as it was being implemented as a part of clinical care in the study setting. The clinic was initially deployed in order to address the unpredictable and fragile transitions for patients with SUD leaving the adult hospital and emergency department (ED), including those with injection-related infections. The model is predicated on the theory that both SUD and the co-occurring disorders that accompany it disrupt the reward, stress, and executive planning neurocircuitry such that transitions in care must be facilitated to improve retention and engagement in care [25]. The deployment of this new model of care provided an opportunity for rigorous evaluation of its effectiveness given that the clinic, founded with limited hospital resources, has a fixed capacity and therefore those patients for whom referral was not possible can serve as a control group. The effectiveness of this new model of care must be evaluated given funding sources are limited and leveraged to support programs that demonstrate improved outcomes. Moreover, evaluation of both the clinical efficacy and potential financial savings of the bridge clinic model will determine the utility of expansion. This manuscript has been written in accordance with Standard Protocol Items: Recommendations for Interventional Trials (SPIRIT) guidelines, shown in more detail in an additional file (see Additional file 1) [26].

### Study design

The BRIDGE trial is a pragmatic, single-center, superiority, randomized, controlled trial beginning 25 November 2019 at Vanderbilt University Hospital in Nashville, TN, USA. As this is a pragmatic trial testing real-world effectiveness, no additional strategies were included to improve adherence and there were no restrictions on concomitant care or other interventions.

### Study sites and period

Vanderbilt University Hospital is an ~800 bed, tertiary care adult hospital. The study commenced on 25 November 2019 and is anticipated to be completed in 18 months.

### Population

The population included in this study are patients admitted for OUD and being considered for MOUD in consultation with the addiction consult service. The inclusion and exclusion criteria are:

*Inclusion criteria*:

- Aged ≥ 18 years
- Active OUD being considered for MOUD evidenced by accepting a transitional prescription for buprenorphine-naloxone or intramuscular naltrexone injection
- Outpatient plans not fixed prior to admission, defined as a patient reporting a prior relationship with an MOUD provider that they intend to continue after discharge, including cash pay practices
- Patient lives in one of the counties that comprise Middle Tennessee, shown in an additional file listing appropriate zip codes (see Additional file 2), in order to ensure a reasonable chance of the patient having regular transportation to the clinic

*Exclusion criteria*:

- Severe, active co-occurring psychiatric disorders that require a higher level of psychiatric care
- Patients for whom methadone maintenance is deemed the best choice of MOUD
- Patients previously randomized in this study
- Patients who previously were referred to the bridge clinic prior to study initiation

### Enrollment and randomization

In the hospital setting, the addiction consult service is notified of potentially eligible patients via an electronic consult order or referral from the general psychiatry service. The addiction consult service is comprised of an addiction psychiatrist, a psychiatric nurse practitioner, a social worker, a nurse case manager, and a recovery coach, with additional fellows and residents rotating through with some regularity. Those patients for whom an order for an addiction consultation is placed are evaluated by one of the psychiatric providers for MOUD. The addiction consult service social worker further screens the patient for inclusion. Once inclusion eligibility is met, the social worker proceeds with randomization via REDCap.

The capacity of the bridge clinic is fixed—the physical clinic occurs all day Friday and we are unable to see more than a set number of new and follow-up patients—so volume is driven by the number of patients referred from the general adult hospital, the adult emergency department, and the psychiatric emergency department. We allocated a set number of intakes per week to emergency services, but only those patients being referred from the general adult hospital are eligible for randomization and enrollment. Due to capacity limitations, the clinic is unable to care for all patients that could be referred. We are leveraging this gap between demand and capacity to use randomization to determine which patients are offered referral from the adult hospital; randomization ensures that selection is unbiased and allows a meaningful comparison of outcomes between those referred and those not referred. Patients deemed eligible for MOUD are randomized to being offered referral to the bridge clinic or to usual care with referral to community MOUD providers. Since patients are enrolled under a waiver of informed consent, the option of declining the trial is not available. Patients can, however, decline the bridge clinic if it is made available to them. During the duration of the trial itself, the bridge clinic is not available outside of the auspices of the study for those persons who were study eligible.

**Fig. 1:**
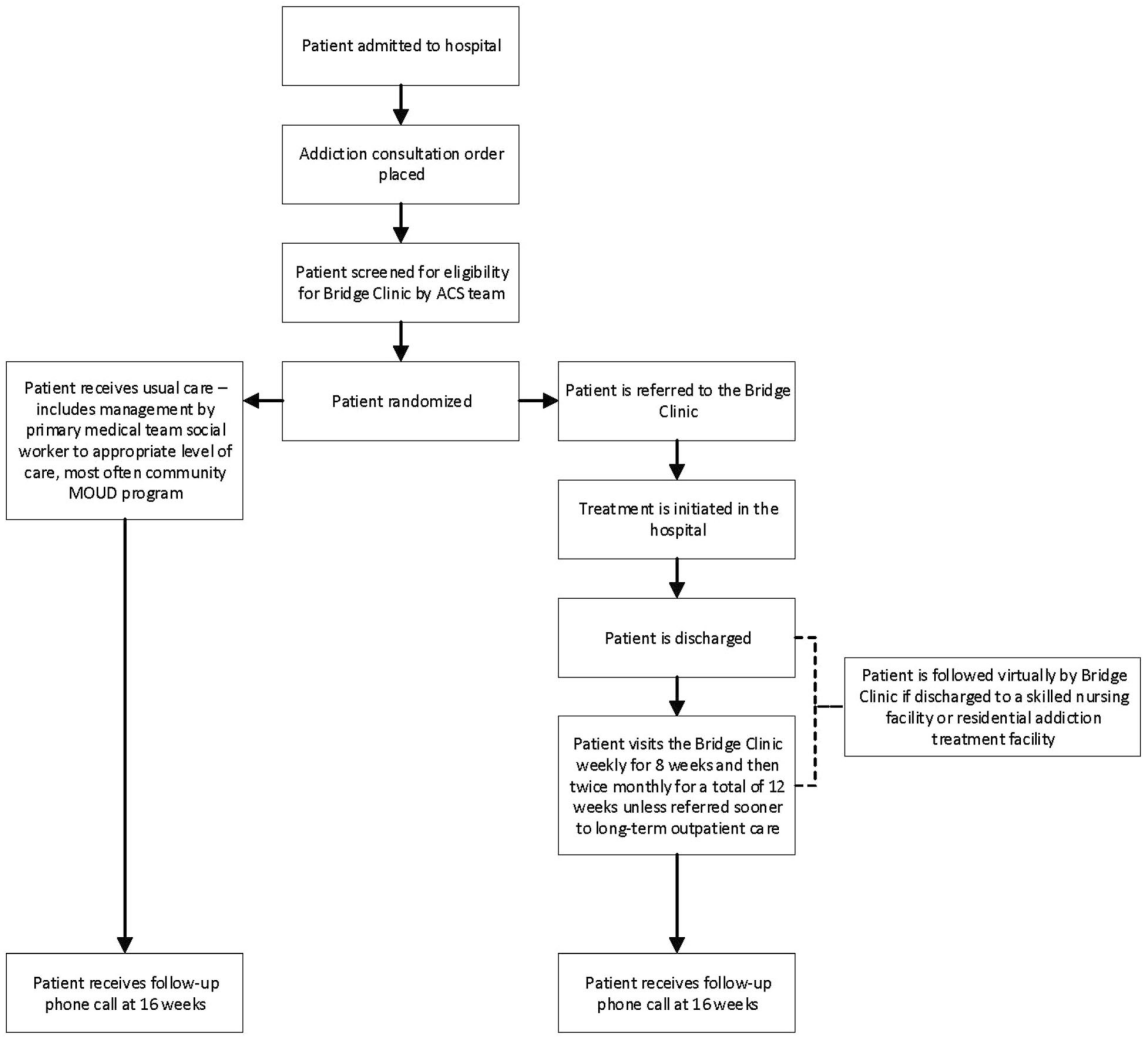
The flow of events for patients screened and randomized into the BRIDGE trial

Randomization occurs using a pre-specified sequence deployed using REDCap’s randomization module [27]. Randomization proceeds regardless of whether the patient prefers to attend a higher level of care (e.g., intensive outpatient, partial hospital program, or residential rehabilitation) on the assumption that these patients will want an outpatient MOUD program after completion of such care. Given the capacity of the clinic and the number of patients eligible, the randomization ratio is set to 1:1, and this is evaluated bi-weekly to maintain the bridge clinic at full capacity. We chose not to stratify randomization based on medical severity because we were unsure a priori how to define severity levels that would meaningfully impact the primary and secondary outcomes. Because this study involves only observational data collection after randomization to bridge clinic or no bridge clinic availability, there are no plans to withdraw participants or discontinue them in the study. A schedule of events is shown in Fig. 1.

### Intervention delivery

The Vanderbilt bridge clinic model includes a team that provides wraparound services for patients with SUD during their post-acute care, regardless of insurance status. The pharmacy provides institutionally grant-funded medication to uninsured patients, and charges (including visit and lab charges) for these patients are written off by the institution. As of September 3, 2020, 57% of the clinic patients were uninsured. The care team is comprised of four different physician specialists (addiction psychiatry, internal medicine, infectious diseases, and pain-anesthesia), a psychiatric nurse practitioner, two licensed social workers, a nurse case manager, and two recovery coaches. All non-medical team members have some background or additional training in the management of addiction and other mental health conditions. These non-medical providers collectively provide tailored case management and other brief psychotherapeutic interventions (e.g., motivational interviewing, education, twelve-step facilitation).

Team members work across addiction treatment settings at our institution, supporting the addiction consult service, the ED, and longitudinal outpatient clinics. The addiction consult service initiates care with patients in the hospital and, for those referred to the bridge clinic, they maintain contact thereafter both remotely and through in-person clinic visits. Patients generally present in-person, though there has been a transition to telemedicine for some patients for brief periods during the COVID-19 pandemic.

Our bridge clinic opened on May 17, 2019, with service one-half day each week in two different locations: one for primary care, psychiatry, and pain-anesthesia providers on Friday mornings, and one for infectious diseases on Friday afternoons. On November 8, 2019, the clinic was relocated to a space that accommodates the entire team all-day Friday, facilitating a traditional medical model in which a medical assistant situates patients in one of seven rooms after collecting intake information, where other providers rotate seeing the patient as needed. This improves continuity of care as it allows multiple providers to see a patient together when there is a need for care coordination.

The bridge clinic primarily cares for patients with OUD who may have any number of co-occurring psychiatric, SUD, and medical issues. Patients are asked to present weekly to the clinic for the first 8 weeks of treatment (assuming they maintain abstinence), then twice monthly. The target period for stabilization and transition to a long-term treatment program is no more than 3 months, though in practice this may be as long as a year if there are challenges to successful transition. We estimate based on early anecdotal experience with the clinic referral process that 75% of new patients scheduled will attend, and those patients will attend on average for eight out of ten visits over the initial 12-week period.

The visit model and provider-patient pairing are designed to be tailored to patient needs within this general framework. At the time of referral into the clinic, the addiction consult service social worker and nurse case manager prioritize pairing patients with a medical provider based on the patient’s co-occurring medical and psychiatric conditions. Because of the flexibility of the model, a medical provider can obtain additional evaluations from other specialty providers in the clinic (e.g., psychiatry provider asking the primary care provider to assess a rash, or infectious diseases provider asking pain-anesthesia to evaluate low back pain) either same-day or at a subsequent visit. Social workers, case managers, and recovery coaches also adjust their pairing over time with patients, though we try to have a social worker and recovery coach follow each patient longitudinally. Medical and non-medical providers use huddles before or after visits to discuss treatment plans for patients, especially patients whose recovery is tenuous and may require referral to a higher level of care. Social work, case management, and recovery coaching staff facilitate referrals to other levels of care and communicate with patients outside of the physical clinic to advance these treatment plans. Permanent transition out of the clinic into appropriate long-term care is addressed as often as every visit, whether the patient requires a higher level of care or not.

Patients are seen for each visit by at least one medical provider and at least one member of the team who can provide psychotherapy, peer support, or case management until they demonstrate 8 weeks of abstinence. The patient provides a urine specimen for toxicology testing at each visit. In rare cases, patients will opt to leave a visit before seeing a non-medical provider, but this is discouraged. After patients have finished seeing the appropriate providers, they are directed from the clinic to the lab and pharmacy. The lab is in the same building and the institutional pharmacy is located just two blocks away. Point-of-care urine drug screening is not currently done but is being developed for implementation along with another planned relocation to a custom-designed facility in 2021. Insured patients can opt to fill medications at an external pharmacy, although this is rare. Prescriptions for controlled substances (e.g., buprenorphine, gabapentin) are refilled at each visit, with 7-day supplies during initial encounters. Prescription-monitoring program entries are reviewed for each patient at each visit.

Usual care is the traditional care provided by the inpatient medical team’s assigned social worker. Patients randomized to usual care are informed by the addiction consult service social workers that the social worker for their primary medical team will be in communication with them about outpatient resources for MOUD and recovery support. An electronic pass-off is completed by the addiction consult service social worker to the primary team social worker, and this pass-off is also documented in the medical record. The primary medical team social worker then meets with the patient for additional assessment and makes referrals appropriate to the individual patient’s transportation and insurance needs. The addiction consult service continues to work with the patient to initiate MOUD and address comorbidity during their hospital admission.

### Data collection

Patient information is documented as a component of usual clinical care and will be extracted from the electronic health record (EHR) for this study. This includes demographic information (i.e., age, gender, race, ethnicity, socio-economic indicators) as well as the length of stay.

Many patients are admitted with infections related to injection drug use and require weeks of treatment with intravenous antibiotics. As part of the BRIDGE trial, we have developed a protocol to assess candidacy for outpatient parenteral antibiotics therapy (OPAT) for patients who are randomized to the bridge clinic for post-acute follow-up care. Information obtained during OPAT candidacy screening is entered into a secure data management system, REDCap, including the history of prior hospital misuse, housing status, telephone access, 24-7 access to a refrigerator for antibiotics storage, presence or absence of SUD in cohabitants, willingness to allow home health services into the home, presence or absence of home support person, and willingness of home support person to confirm the presence of stable housing, to assist with antibiotic administration, and to assist patient with attending medical appointments. These data inform the eligibility of the patient for the OPAT protocol.

Prior to April 2021, only patients randomized to the bridge clinic were potentially eligible for OPAT. In theory, this would favor the bridge clinic in terms of the primary outcome (LOS), though this is not a limitation per se but rather part of the central hypothesis. In April 2021, in response to extreme hospital bed demand related to COVID-19, we adjusted our protocol such that OPAT eligibility took precedence over randomization. At that point, any patient deemed eligible for OPAT would be referred to bridge clinic and not included in the trial.

Part of usual care for patients seen by the addiction consult service and initiated on MOUD also includes a 16-week follow-up phone call, at which time additional information is collected. This call is also conducted for patients randomized to attend the bridge clinic. These data include initial attendance at outpatient MOUD follow-up, self-reported relapse to use of illicit opioids and frequency of use in preceding 30 days, number of overdoses since discharge, cross-over from usual care to bridge clinic or vice versa, and a measure of overall psychological well-being (Schwartz Outcome Scale-10) [28–30].

At the time of the 16-week follow-up, case managers make up to three telephone calls, leaving voicemails if the patient does not respond. Beginning in August 2020 and due to an initially low response rate, each call is accompanied by a text message from recovery coaches on their institutional mobile device or through our institutional patient portal for those patients who prefer it. Case managers attempt to engage additional contacts in the medical record when patients are unable to be reached. Finally, some patients cannot be reached for documented reasons at 16 weeks, such as incarceration or death.

### Primary outcomes

The primary outcome measured is the overall index hospital admission length of stay. Hospital length of stay is an accepted metric in operational health care research. It was chosen in this case in light of the observation that patients with OUD were often mismatched with the treatment setting for two reasons: (1) they were often being kept in the hospital due to a standard of care holding that patients with OUD should not be eligible for ambulatory parenteral antibiotic therapy given the risk of misuse of central venous access devices, despite evidence that this can be done safely [31, 32], and (2) the general medical hospital does not tend to provide a therapeutic environment for patients in early recovery from OUD who are isolated and lack access to behavioral treatments and community mutual help supports. Therefore, reduction in hospital LOS represents an easily measured proxy for improving an outcome that matters to both providers and patients in terms of advancing OUD recovery and providing value to the hospital system.

### Secondary outcomes

The secondary outcomes, all assessed for the 16-week follow-up period, are:

- Linkage to MOUD provider, defined as attending at least one visit with a MOUD provider after discharge, based on self-report at 16-week follow-up call. Such engagement is considered a process measure in the field for engagement correlated with outcome measures related to abstinence at 6 months follow-up [33].
- Self-reported buprenorphine-naloxone (or naltrexone) prescriptions filled, a related process measure
- Readmission rates
- ED visits
- Hospital-free days
- ED-free days
- Recurrent opioid use
- Overall quality of life as measured by the Schwartz Outcome Scale-10 (SOS10) and EQ-5D-5L. We chose to add these additional quality measures given that the OUD literature has often neglected so-called functional outcomes that matter to patients other than abstinence from illicit substances, though certainly abstinence is thought to correlate with improved quality of life [34].
- Overdose
- Mortality
- Cost of care, including total costs for each admission and care resources utilized. Our medical center finance team is able to provide these costs as another useful measure of value provided by the intervention which may capture LOS reduction but also reduction in other costs that the bridge clinic may influence like readmission, substance misuse in the hospital with reinfection, and patients leaving the hospital against medical advice. We will not be able to easily capture costs associated with the use of other medical centers in the region for purposes of this study.

Additional outcomes for patients with infection suitable for outpatient management include:

- New, persistent, or recurrent infection (as defined by a positive culture and/or change in antibiotic regimen)
- Completion of antibiotic therapy
- The number of days from negative blood culture to first hospital discharge

Implementation measurements include acceptance of referral to bridge clinic and cross-over from usual care to bridge clinic and vice versa, as well as any reasons for ineligibility.

### Analysis plan

#### Statistical analysis

Initially, we will characterize participants overall and grouped by study arm using means with standard deviations, medians with interquartile range, and counts with percentages as appropriate. The primary analysis will compare the length of index hospital stay between those offered referral to the bridge clinic and those not offered referral to the bridge clinic on an intent-to-treat basis. We will use a generalized linear model with group assignment as the primary predictor variable, with adjustment for important covariates. Multiple imputation will be used for missing covariates; length of stay will not be missing for any cases. We expect to model length of stay as a continuous outcome. We may choose a proportional hazards model or a gamma generalized linear model with a log-link function if the data are substantially skewed; we do not expect this based on our experience with the length of stay in this patient cohort. We have one primary outcome, and we will use a critical *p*-value of 0.05 to test our primary hypothesis.

Secondary and exploratory analyses will also use the intent-to-treat analysis set. Binary outcomes will be compared between groups using logistic regression, adjusted for covariates. Ordinal outcomes will use a proportional odds model. Costs are expected to be skewed and so a proportional hazards model or a gamma generalized linear model with a log-link function will be chosen.

#### Power calculation

The mean length of stay for patients meeting the inclusion criterion in the 12-to-18-month period before bridge clinic was established was 15 days, ranging from 3 to 42 with a standard deviation of about 15 days. With 358 patients per study arm, we would have 90% power to detect a reduction in length of stay of 3 days, assuming the common standard deviation was decreased to 12.5 days. If the common standard deviation only reduced to 14 days, we would still have 80% power to detect a difference. We therefore selected a sample size of 700, with 350 randomized to each arm. We expected to complete this study within 18 months.

Because of the disruption caused by COVID-19 and lower than expected enrollment, midway through the recruitment period, we re-estimated the sample size based on the experience of patients. Blinded to allocation, we estimated the distribution of length of stay for all enrolled patients. We found the mean length of stay was shorter, at 9 days, with a standard deviation of 11. The length of stay was also decidedly right skewed. Assuming a reduction in length of stay of 1.5 days is meaningful, about 168 patients per group would be required to have 80% power to detect a difference. Given our current accrual rate of four patients per week, if we continue enrolling for 12 months as originally planned, we will obtain a sample size of 336, which is sufficient to have more than 80% power for our primary outcome.

### Data confidentiality, sources, and sharing

Data for this trial will be extracted from the electronic health record and ancillary systems as all information is generated in the usual process of care. Data will be entered into REDCap, which provides a secure environment for the maintenance of the data. Analytical datasets will be stored on secure servers without direct identifiers. On completion of this study, data will be made available on submission of a methodologically sound proposal that is accompanied with appropriate regulatory approvals.

### Presentation of the results

After completion of enrollment and data analysis, the results of the trial will be communicated through manuscript publication. Submission will include public access to the full study protocol and statistical code. Authorship will be based on the International Committee of Medical Journal Editors guidelines (2018), and professional writers will not be used. The results will also be presented at local and national conferences, posted on ClinicalTrials.gov, and used to inform local evidence-based practice committees on care options for hospitalized patients with OUD.

## Discussion

Upon completion, the BRIDGE trial will provide the most comprehensive data to date on the effect of a multispecialty, multidisciplinary transitional clinic focused on continuity of access to MOUD initiated by an addiction consult service during acute care in a general hospital.

Several potential threats to the validity of the trial exist. Because data are being obtained based on charting by practicing clinicians in the electronic health record (EHR), there may be missing data. For the primary outcome, there will be many factors other than the bridge clinic intervention that may impact the length of stay. We expect randomization to mitigate the risk that one arm was disproportionately affected by such factors. Given that all providers are aware of the trial itself and the randomization status of a given patient, it is possible that unconscious or conscious biases will lead to differential treatment of patients based on randomization condition in ways that go beyond the scope of the trial itself. Furthermore, all patients considered for the trial receive addiction consult services, an intervention which may reduce observed effect size by reducing the length of stay compared to hospitalized patients with OUD who do not receive addiction consult services.

Our study is a pragmatic trial, which provides the opportunity to evaluate the effectiveness of an intervention in real-life routine practice conditions [35]. Inherent in this design however is the potential for a lack of external validity. Additionally, with a single-center trial, there is limited ability to generalize the study results. Availability of an addiction consult service is not universally available or feasible in all general hospital contexts.

Other than certain objective measures such as ED visits, readmissions, and costs of care, many secondary outcomes are based on self-report by patients at their 16-week follow-up. There is a high risk of bias as to which patients staff reached to obtain 16-week follow-up data. For example, those that are more successful with their recovery may be more likely to maintain continuous cell phone access and to want to report on their progress. If considerable bias in follow-up rates is observed, we may test the external validity of our findings by repeating our primary analysis using inverse probability weighted for propensity to follow-up.

Additional sources of bias include changes in the availability of community-based care and the ability of all patients to engage in community-based mutual help and other supports as this trial coincided with the COVID-19 pandemic. Many of these societal changes could have affected both randomization groups equally, though a reduction in community MOUD programs as well as transportation and other resources could have disproportionately affected patients in the usual care group. Finally, we did consider the potential influence of patients departing the hospital against medical advice (AMA) in the design and determined that it would likely bias the trial results towards the null. Patients with longer lengths of stay may be more likely to leave AMA and thus reduce the LOS overall. If our hypothesis is supported, this would have a greater influence on the TAU arm when compared with the BRIDGE arm.

Despite the challenges and potential biases, the ongoing pragmatic BRIDGE trial will provide evidence on the effectiveness of proactive linkage to a bridge clinic intervention for hospitalized patients with OUD initiating evidence-based pharmacotherapy in consultation with the addiction consult service.

## Trial status

The status of this trial is currently ongoing with enrollment aimed to be completed in the fall of 2021.

## Supplementary Information

**Additional file 1.** Standard Protocol Items: Recommendations for Interventional Trials (SPIRIT) guidelines, PDF, completed checklist of SPIRIT guidelines

**Additional file 2.** Zip Codes for Inclusion, PDF, complete list of all zip codes of residency considered for inclusion in enrollment

## Data Availability

On completion of the study, investigators external to the research team may request to collaborate on secondary analyses. With appropriate IRB approval and data use agreements in place, de-identified datasets may be released on reasonable request. All statistical code will be made publicly available with the analysis. The investigators plan to publish trial results without assistance from outside professional writers. The investigators have no publication restrictions. Materials that are shared with patients as part of the bridge clinic program are under continuous development and are available from the corresponding author upon request.

## Abbreviations

EHR: Electronic health record
ED: Emergency department
MOUD: Medication for opioid use disorder
OUD: Opioid use disorder
OPAT: Outpatient parenteral antibiotics therapy
SOS-10: Schwartz Outcome Scale-10
SPIRIT: Standard Protocol Items: Recommendations for Interventional Trials
SUD: Substance use disorder
