# Supplementary material for: Bridging Recovery Initiative Despite Gaps in Entry (BRIDGE): study protocol for a randomized controlled trial of a bridge clinic compared with usual care for patients with opioid use disorder
Source: Trials. 2021 Oct 30;22:757. doi: 10.1186/s13063-021-05698-4 (PMC8556830; doi:10.1186/s13063-021-05698-4)

Additional file 2

Nashville-Davidson--Murfreesboro--Franklin Metro Area, Tennessee: 113 Zip Codes			
37010 (Adams)	37069 (Franklin)	37145 (Pleasant Shade)	37201 (Nashville)
37013 (Antioch)	37072 (Goodlettsville)	37146 (Pleasant View)	37203 (Nashville)
37014 (Arrington)	37073 (Greenbrier)	37148 (Portland)	37204 (Nashville)
37015 (Ashland City)	37074 (Hartsville-Trousdale)	37149 (Readyville)	37205 (Nashville)
37016 (Auburntown)	37075 (Hendersonville)	37150 (Red Boiling Springs)	37206 (Nashville)
37022 (Bethpage)	37076 (Hermitage)	37151 (Riddleton)	37207 (Nashville)
37025 (Bon Aqua)	37080 (Joelton)	37153 (Rockvale)	37208 (Nashville)
37026 (Bradyville)	37082 (Kingston Springs)	37165 (Slayden)	37209 (Nashville)
37027 (Brentwood)	37083 (Lafayette)	37167 (Smyrna)	37210 (Nashville)
37029 (Burns)	37085 (Lascassas)	37172 (Springfield)	37211 (Nashville)
37030 (Carthage)	37086 (La Vergne)	37174 (Spring Hill)	37212 (Nashville)
37031 (Castalian Springs)	37087 (Lebanon)	37179 (Spring Hill)	37213 (Nashville)
37032 (Cedar Hill)	37090 (Lebanon)	37181 (Vanleer)	37214 (Nashville)
37033 (Centerville)	37098 (Lyles)	37184 (Watertown)	37215 (Nashville)
37035 (Chapmansboro)	37115 (Madison)	37186 (Westmoreland)	37216 (Nashville)
37036 (Charlotte)	37118 (Milton)	37187 (White Bluff)	37217 (Nashville)
37037 (Christiana)	37122 (Mount Juliet)	37188 (White House)	37218 (Nashville)
37046 (College Grove)	37127 (Murfreesboro)	37189 (Whites Creek)	37219 (Nashville)
37048 (Cottontown)	37128 (Murfreesboro)	37190 (Woodbury)	37220 (Nashville)
37049 (Cross Plains)	37129 (Murfreesboro)	38401 (Columbia)	37221 (Nashville)
37051 (Cumberland Furnace)	37130 (Murfreesboro)	38451 (Culleoka)	37228 (Nashville)
37055 (Dickson)	37132 (Murfreesboro)	38454 (Duck River)	37240 (Nashville)
37057 (Dixon Springs)	37135 (Nolensville)	38461 (Hampshire)	37243 (Nashville)
37060 (Eagleville)	37137 (Nunnelly)	38474 (Mount Pleasant)	37246 (Nashville Elec Serv)
37062 (Fairview)	37138 (Old Hickory)	38476 (Primm Springs)	
37064 (Franklin)	37140 (Only)	38482 (Santa Fe)	
37066 (Gallatin)	37141 (Orlinda)	38487 (Williamsport)	
37067 (Franklin)	37143 (Pegram)	38547 (Brush Creek)	
		38552 (Chestnut Mound)	
		38560 (Elmwood)	
		38563 (Gordonsville)	
		38567 (Hickman)	
		38569 (Lancaster)	

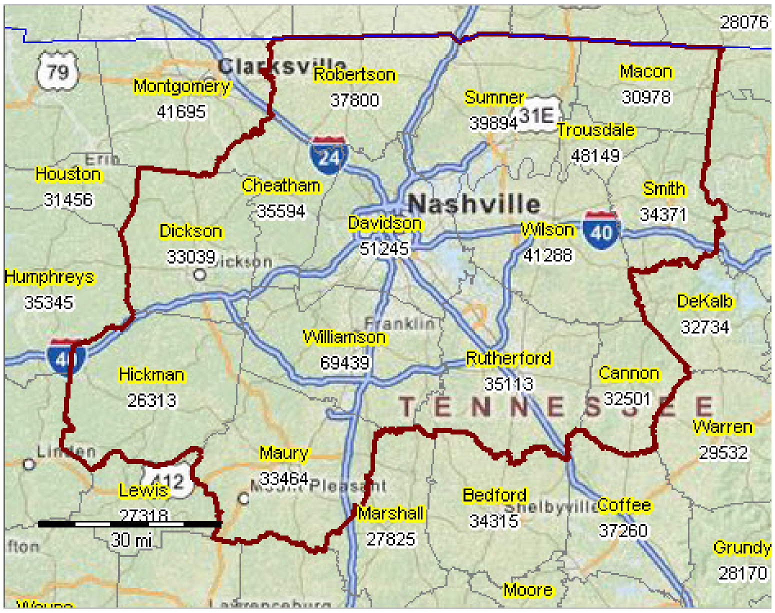

Supplement: Supplementary file 2 — Additional file 2. Zip Codes for Inclusion, PDF, complete list of all zip codes of residency considered for inclusion in enrollment [file 13063_2021_5698_MOESM2_ESM.pdf]
